# PET Imaging of Tau Pathology in Alzheimer’s Disease and Tauopathies

**DOI:** 10.3389/fneur.2015.00038

**Published:** 2015-03-09

**Authors:** Olga G. James, P. Murali Doraiswamy, Salvador Borges-Neto

**Affiliations:** ^1^Division of Nuclear Medicine, Department of Radiology, Duke University, Durham, NC, USA; ^2^Department of Psychiatry and Duke Institute for Brain Sciences (DIBS), Duke University, Durham, NC, USA; ^3^Division of Geriatrics, Department of Medicine, Duke University, Durham, NC, USA

**Keywords:** tau proteins, dementia, PET neuroimaging, Alzheimer’s disease, tau pathologies

Alzheimer’s disease (AD) is a major public health problem affecting nearly 35 million people worldwide with numbers projected to rise to 115.4 million by 2050. Medicare and Medicaid cost of AD is anticipated to increase fourfold to approximately $ 600 billion in the next 40 years. AD is the only cause of death among the top 10 causes that has no prevention or cure (1, 2). AD causes are not fully known and clinical drug trials have a greater than 90% failure rate. There is an urgent need to find accurate methods of early detection as well as effective therapies before patients with AD develop significant brain damage.

Clinical diagnosis of AD remains a challenge since there are no validated tests for an affirmative diagnosis and dozens of conditions can mimic it. Hallmarks of AD brain are numerous neurons with neurofibrillary tangles of paired helical filaments (PHFs), straight filaments, and extracellular deposits of β-amyloid as the major component of senile (neuritic) plaques in the brain. Definitive diagnosis of AD is made only after an autopsy or biopsy. While AD is the most common cause of dementia in the elderly, accounting for approximately 50–70% of cases, many other causes of dementia can mimic and overlap with AD clinical presentation. Vascular dementia, mixed dementia of vascular and Alzheimer’s etiology, dementia with Lewy bodies (DLB), and frontotemporal dementias are some examples with overlapping clinical and diagnostic features.

Clinical diagnosis of probable AD is currently made by excluding other causes using history, exam and labs, structural imaging, and cognitive testing. However, their accuracy is suboptimal. For example, a clinical-autopsy correlative study of more than 900 cases seen at leading academic memory centers found that some 40% of patients clinically diagnosed with non-AD dementia had postmortem histopathology consistent with AD (3). Likewise, nearly 30% of patients, who were thought to have AD, clinically do not meet postmortem pathologic criteria for AD (3). In recent clinical drug trials of mild to moderate AD, it is also estimated that about 30% of patients did not have AD pathology thus making it difficult to determine if failures were due to ineffective drug versus diagnostic errors (4). There is a call for the development and integration of pathologic biomarkers into routine clinical evaluation to establish revised diagnostic criteria for clinical and preclinical AD. Such markers may not only assist with an accurate affirmative diagnosis of AD and disease staging, but may also accelerate drug development (5).

Molecular imaging, specifically positron emission tomography (PET), is a promising modality for early detection and disease staging in Alzheimer’s patients. Recently approved amyloid PET tracers (e.g., F-18 florbetapir, F-18 flutemetamol) can detect cortical fibrillary β-amyloid (6). A negative amyloid scan substantially decreases the odds of a person having AD and can impact clinical decision making or treatment. In at-risk subjects, a positive amyloid scan is associated with a threefold greater risk of converting to dementia (7). The limitations of amyloid imaging are that amyloid plaques by themselves are insufficient for a positive diagnosis of AD. Thus, the advent of a PET taupathology tracer may serve as a complimentary tool to aid in affirmative diagnosis, as well as in disease staging. Given the number of tau-based therapies being developed, a tau-PET tracer would also allow for a selection of pathology-positive individuals and monitor the effectiveness of therapy.

## Pathophysiology of Tau in AD

Tau proteins result from a single microtubule-associated protein tau (MAPT) gene (chromosome 17q21) in humans and were discovered in 1975. Healthy neurons contain microtubules, which serve as a support structure and guide nutritional supplies. Tau protein binds to the microtubules, promotes their assembly, and stabilizes them. There are six isoforms of tau protein in human brain tissue, referred to as τ3L, τ3S, τ3, τ4L, τ4S, and τ4. Each has a distinguishing feature, i.e., number of binding domains repeats (R), of 31 or 32 amino acids in the C-terminal part of tau protein and one (1N), two (2N), or no inserts of 29 amino acids each in the N-terminal portion of the molecule. Three of the tau proteins (τ3L, τ3S, τ3) have three (3R) binding domains repeats and the rest of them (τ4L, τ4S, τ4) have four (4R) binding domains repeats. Chemical changes (namely hyperphosphorylation) occur in tau protein in AD. It is theorized that in a hyperphosphorylated state, they begin to pair up with other threads of tau into PHFs and tangle together, causing disintegration of microtubules, collapse of neuron’s transport system, and formation of extremely insoluble aggregates. These changes are presumed to disrupt neuronal communication and lead to cell death (8).

All six of the τ isoforms are identified in a hyperphosphorylated state in PHFs in the AD brain. There is approximately a four to eightfold higher level of phosphorylated tau protein found in the AD brain compared to that of age-matched healthy brains. Alzheimer’s staging in postmortem brains by Heiko and Eva Braak showed that neurofibrillary tangle, neuropil thread, and neuritic plaque distribution varied widely within cortical architectonic units and from one individual to another. Similar to amyloid plaques, neurofibrillary tangles and neuropil threads showed a characteristic distribution pattern of growth in six stages (see Table S1 in Supplementary Material) from entorhinal cortex to hippocampus to neocortex (9, 10).

Neurofibrillary tangles are generated intracellularly. However, when neurons die, the only neurofibrillary tangles remains are “ghost tangles” which are localized extracellularly. Since many neurons die and leave “ghost tangles,” they are a common finding in AD patients which can occur in preclinical stages (10, 11). Therefore, the ability to identify and evaluate the severity of tau pathology in the brain may further assist affirmative diagnosis of AD and disease progression staging (possibly even after β-amyloid deposition plateaus)and offer evaluation of potential anti-tau treatment efficacy.

While neurofibrillary tangles are known as relatively specific markers for AD, other forms of aggregated tau abnormalities are identified among different forms of dementia, such as Pick bodies in Pick’s disease, globose tangles in progressive supranuclear palsy (PSP), and chronic post-traumatic encephalopathy (CTE).

## PET Tau Tracers

The ideal PET tau tracer would have a strong selective binding potential to PHFs and phosphorylated tau over β-amyloid (β-amyloid has higher concentration in diseased brains), high permeability of blood–brain barrier, low metabolism, and low non-tau (non-target) binding to other CNS receptors and tissues (e.g., white matter) (12–15). To our knowledge, at least seven tau pathology PET tracers have been developed and used in clinical studies: C-11 PBB3, F-18 THK-523, F-18 THK-5105, F-18 THK-5117, F-18 T808, F-18 FDDNP, and F-18 T807. Not all of these agents provide the desired specific affinity to tau tangles. For instance, F-18 FDDNP demonstrates binding to both β-amyloid and tau pathology (it was not designed as a specific tau tracer but as a tracer for both pathologies). Another tau-targeting radiotracer, THK-523, had an uptake pattern in AD patients that was not distinguishable from healthy controls (12).

Preliminary data for another agent from this group, F-18 TKH5105, an arylquinoline derivative, have shown that this probe selectively binds to pathological PHF tau-deposition in living patients with AD, and may differentiate diseased brains from healthy controls. Patients with AD had high retention of F-18 TKH5105 in the temporal cortex, an area that is known to have high densities of neurofibrillary tangles in AD population, compared to the cerebellum. Healthy controls’ uptake in the inferior temporal cortex was identical to the activity in the cerebellum. TKH 5105 was also observed to have *in vitro* binding to glial tau pathology in corticobasal degeneration and PSP. Further studies are necessary to determine if this particular agent will be useful for imaging tauopathies other than AD. The researchers did not report any toxic events and observed a rapid entry of this agent into the gray matter areas (13).

**Figure 1:**
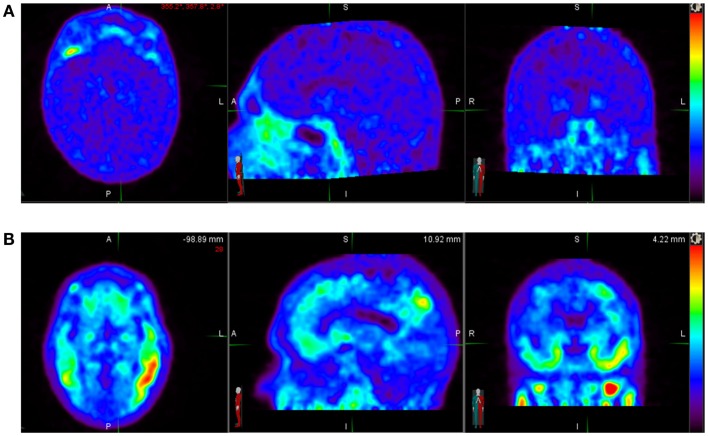
**(A)** Normal ^18^F-AV-1451 PET study illustrating a tau-PET scan from an elderly cognitively normal subject (MMSE score of 30). No abnormal uptake is demonstrated above diffuse background activity. **(B)** Abnormal ^18^F-AV-1451 PET study illustrating a tau-PET scan images of a subject with mild Alzheimer’s disease (MMSE score of 22). Unlike an individual with normal cognition, there is abnormally increased radiotracer uptake distributed in the temporal lobes bilaterally. *Source*: Duke University.

^18^F-AV-1451 [(F-18)T807] is in Phase 2 development as a diagnostic PET tracer for *in vivo* imaging of tau aggregate pathology in patients with AD and related neurodegenerative diseases characterized by the presence of tau pathology (Figure 1). Hence, we discuss it here as a prototypic tracer. It is an F-18-labeled small molecule that demonstrates high selective binding and affinity (15 nM *K*_d_ in brain slices and 0.7 nM in purified PHF-tau) to tau protein aggregates and relatively spares normal monomeric tau proteins in *ex vivo* human brain. One preclinical investigation of ^18^F-AV-1451 showed that brains with significant tau tangles burden or in combination with β-amyloid (“tau-rich” brains) had increased uptake of the radiotracer in the gray matter. Healthy controls and brains mostly containing β-amyloid (“tau-poor” brains) did not demonstrate such findings.

The results suggested that ^18^F-AV-1451 selectively bound to PHFs, and has very weak or no affinity to the β-amyloid accumulation: an approximately 29 times greater difference was found between binding to tau aggregates compared to β-amyloid in the gray matter of AD brains (14).

This radiopharmaceutical decays via positron emission to O-18 with half-life of 110 min. ^18^F-AV-1451 is administered intravenously with a dose of up to 10 mCi (370 MBq) for a whole body effective dose of 8.92 mSv. Several preliminary studies with AV-1451 have been presented at scientific meetings and several Phase 2 trials in AD, CTE, and other tauopathies are ongoing. To date, several dozen AD and control subjects have been studied (Figure 1) without any significant serious safety concerns. ^18^F-AV-1451 had no significant distribution in the normal brain showing only a diffuse pattern of background activity in healthy controls, unlike brain in patients with high probability of AD, with regional distinct areas of uptake in the gray matter (14). In another analyses of 40 subjects with normal or abnormal cognition, worse memory performance was associated with greater PET tau binding in the entorhinal cortex.

Further data from multicenter studies will soon clarify the clinical value of combining information from both amyloid and tau-PET scans. The cost of performing two PET scans (amyloid and tau scan) and risks (radiation exposure, incorrect interpretation) will need to be weighed against the potential benefits. Tau imaging is also likely to be included in several Phase 2 and 3 drug trials of tau-targeted therapeutics in development and such studies will inform us about its value as a therapeutic biomarker.

In summary, tau-PET imaging represents a significant new advance for the field and it is hoped that the combination of tau positive and amyloid positive PET scans, along with the clinical presentation, may in the future move us closer to an affirmative *in vivo* diagnosis of AD. Tau scans may also enhance diagnosis and testing of tau-based therapies in FTD and CTE, and the results of controlled trials testing its effects on outcomes and cost of care will more definitively guide its role in the clinical work up of people with memory problems.

## Summary

^18^F-AV-1451 is one of several new investigational PET radiopharmaceuticals under study for *in vivo* imaging of tau pathology in patients with AD. The primary data show its high selectivity for binding to tau protein aggregates vs. β-amyloid in human brain tissue. Also, the first reports show its favorable kinetics, rapid delivery into the brain, and clearance from the white matter. It demonstrates very low non-specific binding in white matter as well as cortical gray matter of healthy subjects. Subjects with different dementia severity showed different patterns of radiotracer accumulation both in density and anatomical spread, potentially reflective of pathological Braak staging of tau deposit patterns.

## Conflict of Interest Statement

Dr. P. Murali Doraiswamy has received research grants (through Duke University) from Avid, Alzheimer’s drug Discovery Foundation, Lilly, Elan, Novartis, Neuronetrix, Medivation, Janssen, Pfizer, and Forum. He has received advisory or speaking fees from Accera, Anthrotonix, Muses Labs, Avid, AstraZeneca, Abbvie, Baxter, Cognoptix, Lundbeck, Takeda, Piramal, Genomind, Sonexa, Shire, Targacept, T3D, Grifols, Neuronetrix, TauRx, Medivation, Danone, and Neurocog Trials. He owns shares in Maxwell Health, Muses Labs, and Adverse Events Inc whose products are not discussed here. He is an investigator in several Amyloid and Tau imaging studies sponsored by industry, NIH, and DOD. Dr. Olga G. James served as a trainer for the Amyvid reader training program offered by Eli Lilly and Avid Radiopharmaceuticals. She is also a co-investigator on Avid/Lilly trials of amyloid and tau tracers. Dr. Salvador Borges-Neto serves as a co-investigator on Avid/Lilly trials of amyloid and tau tracers.

## Supplementary Material

The Supplementary Material for this article can be found online at http://www.frontiersin.org/Journal/10.3389/fneur.2015.00038/full
