# Supplementary material for: PET Imaging of Tau Pathology in Alzheimer’s Disease and Tauopathies
Source: Front Neurol. 2015 Mar 9;6:38. doi: 10.3389/fneur.2015.00038 (PMC4353301; doi:10.3389/fneur.2015.00038)
Supplement: Supplementary file 1 [file Table_1.PDF]

Table:1 Braak Pathologic staging of PHFs

**Transentorhinal I-II**		**Limbic III-IV**		**Isocortical V-VI**	
**I**	**II**	**III**	**IV**	**V**	**VI**
First neurofibrillary tangles (NFTs) in the transentorhinal region.	Additional NFTs in the pre-alpha layer of the entorhinal cortex.	Further NFTs in the pri-alpha layer of the entorhinal cortex and in the CA1 (*Cornu Ammonis)*/subiculum region of the hippocampal formation. Initial NFTs in the basal temporal neocortex.	NFTs in the temporal neocortex including the superior temporal gyrus.	NFTs in secondary and tertiary cortical fields. Primary cortex areas such as the primary visual cortex are spared.	NFTs all over the brain including primary cortex fields.
