# Supplementary material for: PET Imaging of Tau Pathology in Alzheimer’s Disease and Tauopathies
Source: Front Neurol. 2015 Mar 9;6:38. doi: 10.3389/fneur.2015.00038 (PMC4353301; doi:10.3389/fneur.2015.00038)
Supplement: Supplementary file 2 [file Table_2.PDF]

Table 2: Tau pathology isoforms distribution in different diseases [12]

Disease	Tau Isoform	Pathological findings
Alzheimer's	3R and 4R	Neurofibrillary tangles<br>Neuropil threads
Progressive Supranuclear palsy	4R	Globose tangles<br>Tuft shaped astrocytes
Pick's disease	3R	Pick's bodies
Cortical basal degeneration	4R	Astrocytic plaques<br>Argyrophilic threads<br>Coiled body<br>Ballooned neurons
